# Putative Self-Organizing Human Corneal Organoids Recapitulate Human Corneal Architecture and Cellular Diversity

**DOI:** 10.3390/bioengineering13050518

**Published:** 2026-04-29

**Authors:** Timothy A. Blenkinsop, Anne Z. Eriksen

**Affiliations:** 1 Department of Ophthalmology, Icahn School of Medicine at Mount Sinai, New York, NY 10029, USA; 2 Department of Health Technology, Technical University of Denmark, 2800 Kongens Lyngby, Denmark; annze@dtu.dk

**Keywords:** corneal organoids, human embryonic stem cells, SEAM protocol, single-cell RNA sequencing, limbal stem cells, corneal epithelium, tissue engineering, regenerative medicine

## Abstract

**Background:** Corneal organoids derived from pluripotent stem cells have emerged as powerful tools for studying corneal development, disease modeling, and regenerative medicine applications. While previous protocols have successfully generated corneal tissue structures, there remains a need for three-dimensional models that recapitulate the complex cellular architecture and diversity of native human cornea. **Methods:** We developed a modified spontaneous three-dimensional corneal organoid model using human embryonic stem cells (hESCs) through an adapted Self-formed Ectoderm Autonomous Multi-zone (SEAM) protocol. hESCs were cultured as spheroids in ultra-low-binding plates under normoxic conditions and differentiated over 7–8 weeks. Organoids were characterized using immunofluorescence staining for corneal-specific markers and single-cell RNA sequencing to assess cellular composition and gene expression patterns. **Results:** Approximately 20% of organoids developed transparent regions characteristic of corneal tissue by day 30 of differentiation. Immunofluorescence analysis revealed spatially organized expression of corneal markers, including ZO-1 and E-cadherin in the outermost epithelial layers, P63α-positive putative limbal stem cells at the epithelial–stromal interface, vimentin-positive stromal cells in the interior, and laminin-1 deposition that suggests Bowman’s membrane formation. The organoids expressed cornea-specific keratins (K3, K12, and K15) and the master regulator PAX6 in appropriate cellular compartments. Single-cell RNA sequencing identified 18 distinct cell clusters, including three corneal epithelium subclusters with differential expression of MUC16, KRT12, and ΔNp63α, two stromal populations with distinct inflammatory profiles, and a corneal endothelium cluster. Transcriptomic analysis confirmed expression of key corneal genes, including AQP3, CDH1, multiple keratins, mucins, and extracellular matrix components (HAS2, CD34, CD44, COL8A1, and KERA). **Conclusions:** This three-dimensional spheroid-based putative corneal organoid model successfully recapitulates the multilayered architecture and cellular diversity of human cornea, including stratified epithelium, putative limbal stem cells, stroma, and endothelium in spatially appropriate arrangements. The model demonstrates molecular signatures consistent with native corneal tissue and provides a valuable platform for studying corneal development, disease mechanisms, and potential therapeutic applications. Future optimization to improve organoid formation efficiency and functional maturation will enhance the utility of this system for both basic research and translational medicine.

## 1. Introduction

Tissue engineering of corneal structures has been applied for corneal diseases as an approach for repair [1]. Organoids represent a groundbreaking advancement in biomedical research, comprising three-dimensional, self-organizing cellular structures derived from stem cells that recapitulate the architecture, functionality, and cellular heterogeneity of native organs in vitro [2]. These miniaturized organ models have profoundly reshaped the landscape of disease modeling by enabling the study of human-specific pathologies in a physiologically relevant context, facilitating high-throughput drug screening and supporting personalized medicine approaches while reducing reliance on animal models [3].

In the field of ophthalmology, organoids have emerged as powerful tools to investigate ocular development and disorders, with corneal organoids specifically offering insights into the cornea—a transparent, avascular tissue essential for refraction and barrier function in the eye. The development of corneal organoids traces back to early efforts in generating ocular structures from pluripotent stem cells. The first reported human corneal organoids were described in 2017, derived from induced pluripotent stem cells (iPSCs) through sequential differentiation protocols that yielded multilayered structures mimicking the epithelial, stromal, and endothelial layers of the developing cornea [4]. This milestone was closely followed by additional protocols for mini-corneal organoids later that year, further refining methods to produce complex, three-dimensional corneal analogs [5].

Building on foundational work in broader organoid technologies, such as intestinal organoids established in 2009 [6], these corneal models have evolved rapidly, incorporating single-cell transcriptomics to characterize developmental trajectories and cellular fates over time [7]. To date, corneal organoids have found diverse applications in research and preclinical studies. They serve as robust platforms for modeling corneal diseases, such as keratoconus, where single-cell RNA sequencing has revealed dysregulated cell fates and integrated responses across epithelial, stromal, and endothelial compartments [8]. In wound healing investigations, organoids integrated into cornea-on-a-chip systems have enabled the study of epithelial repair mechanisms and therapeutic interventions [9]. Additionally, they facilitate drug toxicity testing and efficacy evaluations, ex vivo studies of corneal epithelial stem cell therapies for conditions like dry eye syndrome [10], and functional genomic analyses during early corneal development [11].

Emerging translational applications include the generation of iPSC-derived corneal epithelial sheets for clinical transplantation in limbal stem cell deficiency and organoids as potential grafts for treating corneal injuries, highlighting their promise in regenerative medicine [12,13]. A model was developed, the Self-formed Ectoderm Autonomous Multi-zone (SEAM), which exhibits cells from both anterior and posterior cell types [14]. This model begins two-dimensionally, then over the course of weeks, begins to grow in three dimensions [15]. A modified version of SEAM was effective at characterizing the corneal tropism of SARS-CoV-2 [16,17] and modified to specify iris muscle cells [18]. We hypothesized that a modified version of this method may enable complex corneal 3D architecture if initiated as a spheroid. The model generates 3D organoids that become specified over the course of weeks. By day 30, they exhibit cell fate specification of corneal cells in natively arranged orientation. In the present study, we put forward this modified putative corneal organoid model, which includes protocol details, selection criteria, and initial characterization.

## 2. Methods

### 2.1. Human Embryonic Stem Cells Culture and Maintenance

This study was approved by the Embryonic Stem Cell Research Oversight (ESCRO) Committee of Mount Sinai (AGR-30186). Human embryonic stem cell (hESC) line H9 was obtained from WiCell. hESCs were cultured and maintained on irradiated MEFs in Dulbecco’s Modified Eagle Medium: Nutrient Mix F-12 (DMEM/F12, Life Technologies, Carlsbad, CA, USA), containing 20% knockout serum replacement (KSR, Life Technologies) and supplemented with 1× L-Glutamine (Life Technologies), 1× MEM Non-Essential Amino Acids Solution (Life Technologies), 1× Penicillin–Streptomycin (10,000 U/mL, Life Technologies), and 0.1 mM 2-mercaptoethanol (Sigma, St. Louis, MO, USA). Before use, 10 ng/mL FGF-basic (GIBCO, Grand Island, NE, USA) was freshly added to the medium. Prior to differentiation, hESCs were seeded onto Matrigel-coated dishes (Corning, New York, NY, USA) in mTeSR1 (STEMCELL Technologies, Vancouver, BC, Canada) media for at least three passages.

### 2.2. 3D SEAM Corneal Cultures

H9 ESC passage 39 were split from Matrigel support following the ReLeSR protocol and suspended in mTeSR media with 40 nM Thiazovivin + 2.5% Matrigel (5% from 1:1 Matrigel:DMEM:F12 stock) and seeded at 3000 cells/well in an ultra-low-attachment 96-well plate. After 2 days, the differentiation was initiated by changing half the media volume to SEAM differentiation medium: GMEM (Life Technologies), 10% knockout serum replacement (KSR, Life Technologies) supplemented with 1× L-Glutamine (Life Technologies), 1× MEM Non-Essential Amino Acids Solution (Life Technologies), 1× Penicillin–Streptomycin (10,000 U/mL, Life Technologies), Sodium Pyruvate (100 mM, Life Technologies) and 55 μm 2-mercaptoethanol (Sigma). Medium changes were performed three times per week throughout the differentiation, in which half of the medium was changed for the first week of differentiation, after which full medium changes were made. Between four and seven weeks of differentiation, SEAM structures were mature and pigmented. At seven weeks, they were analyzed and/or harvested. Between days 32 and 46, corneal maturation was enhanced by changing the media to DM:CnT-PR (CellnTec, Bern, Switzerland) (1:1) supplemented with 10 ng/mL keratinocyte growth factor (KGF) and 40 nM Thiazovivin. From day 48 onwards, the media was changed to a maintenance media consisting of DMEM:F12, 2% B-27 supplement, 10 ng/mL KGF and 2 μm Thiazovivin.

### 2.3. Immunofluorescence Staining

The organoids were fixed in 4% paraformaldehyde (PFA), washed with Phosphate-buffered saline (PBS) three times for 5 min. Organoids were then prepared for cryo-sectioning, incubating in 10% sucrose overnight, before changing to 30% sucrose and incubating for an additional 36 h. The organoids were frozen in blocks of Tissue-Tek^®^ O.C.T. Compound (SAKURA, Chikuma-City, Japan) and cut into 14 μm sections. The sections were put in superfrost glass slides (Thermo Fisher, Waltham, MA, USA) and incubated with PBS containing 5% goat serum and 0.3% Triton X-100 for 1 h to block non-specific reactions. They were then incubated with the antibodies shown in Supplementary Table S1 at 4 °C overnight. Slides were then washed twice with PBS for 5 min and incubated with a 1:200 dilution of Alexa Fluor 488-, 568-, 647-conjugated secondary antibodies (Life Technologies) for 1 h at room temperature. Counterstaining was performed with DAPI before mounting with ProLong Gold Antifade Mount (Thermo Fisher Scientific, Waltham, MA, USA), then imaging was performed on an inverted epifluorescent microscope (Leica, Wetzlar, Germany).

### 2.4. Single-Cell RNA Sequencing

At day 153, one organoid was digested with 2.5 mg/mL collagenase II to form a single-cell suspension. Viability of single cells was assessed using Trypan Blue staining, and debris-free suspensions of >80% viability were deemed suitable for single-cell RNA Seq. The single suspension was processed for 10× scRNAseq using the Chromium Single-Cell 5′ Library and Gel Bead Kit v1.1 using an input of ~10,000 cells, following the manufacturer’s protocols.

The sample was sequenced on the Novaseq 6000 Illumina sequencer (Illumina, San Diego, CA, USA) with S4 flow cell (100/paired end reads), targeting a depth of 50,000–100,000 reads per cell using v3 chemistry at the genomics core facility at Mount Sinai or on an in-house Nextseq 500 High-Capacity flow cell. Fastq files were generated using Cell Ranger Single-Cell Software Suite (v3.1) and aligned to the grch38 reference genome. Downstream analyses and graph visualizations were performed in the Seurat R package (v. 3.1.2). Briefly, we removed cells with unique gene counts greater than 7500 (potential doublets) and less than 200, as cell with more than 30% mitochondrial genes. The remaining cells were normalized by a global-scaling normalization method with the default scale factor. Linear dimensional reduction was performed by PCA, following which clustering was performed. The results were visualized using Uniform Manifold Approximation and Projection (UMAP) plots for dimension reduction. Violin and individual gene UMAP plots were generated using the Seurat R package. Cluster annotation was guided by manual gene expression notation, which was complemented by Enrichr gene set enrichment analysis (GSEA), using the top 100 differentially expressed genes for each cluster. Jensen TISSUES (Copenhagen, Denmark) text mining provided the association between genes and human tissues, the Mouse Gene Atlas from BioGPS (Version 2023) was used for cell type specifications and gene ontologies were generated using the GO biological process 2018 terms.

## 3. Results

**Figure 1:**
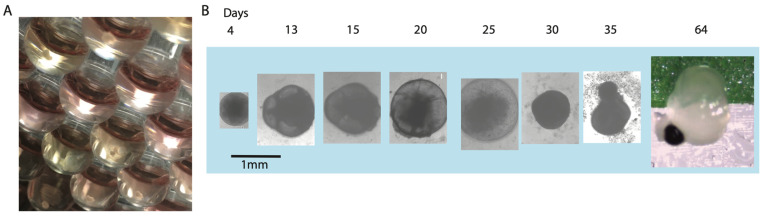
Representative images of the temporal maturation of SEAM 3D corneal organoids. (**A**) Image of 20-day-old corneal organoids from the underside of 96-well, rounded-bottom, ultra-low-attachment plates. (**B**) Phase and color images of corneal organoid examples from timepoints from days 4 to 64 during differentiation.

Pluripotent stem cells were used to differentiate into corneal organoids according to the described Protocol (N-3). After the 7-week time point of culturing, the 3D corneal organoid cultures were evaluated for their physiological properties (Figure 1A). We designated a putative corneal organoid when >50% of the organoid exhibits transparency. In the three differentiations, we began with 96 spheroids or a total of 288. Of those, 54 (19.4%) of initial spheroids developed into putative corneal organoids, with large transparent liquid-filled compartments. These segments may only be a small section, or they may encompass the complete organoid. The organoids start out as small spheres that begin forming cysts around day 13 (Figure 1B). Around day 20, pigmented spots develop on the organoids. From days 25 to 30, a de-swelling of cysts was observed, likely due to the development of more extracellular proteins and an increase in the thickness of the epithelial layer. The mature organoids demonstrated pigmented regions and transparent, cornea-like regions. The transparency was determined by removing them from their well and placing them in a PBS droplet on top of colored tape. If the colored tape was easily observed with little cloudiness, the organoid was deemed a putative corneal organoid and accepted for further analysis.

### 3.1. Immunofluorescent Characterization of D70–80 Organoids Using Corneal Markers

**Figure 2:**
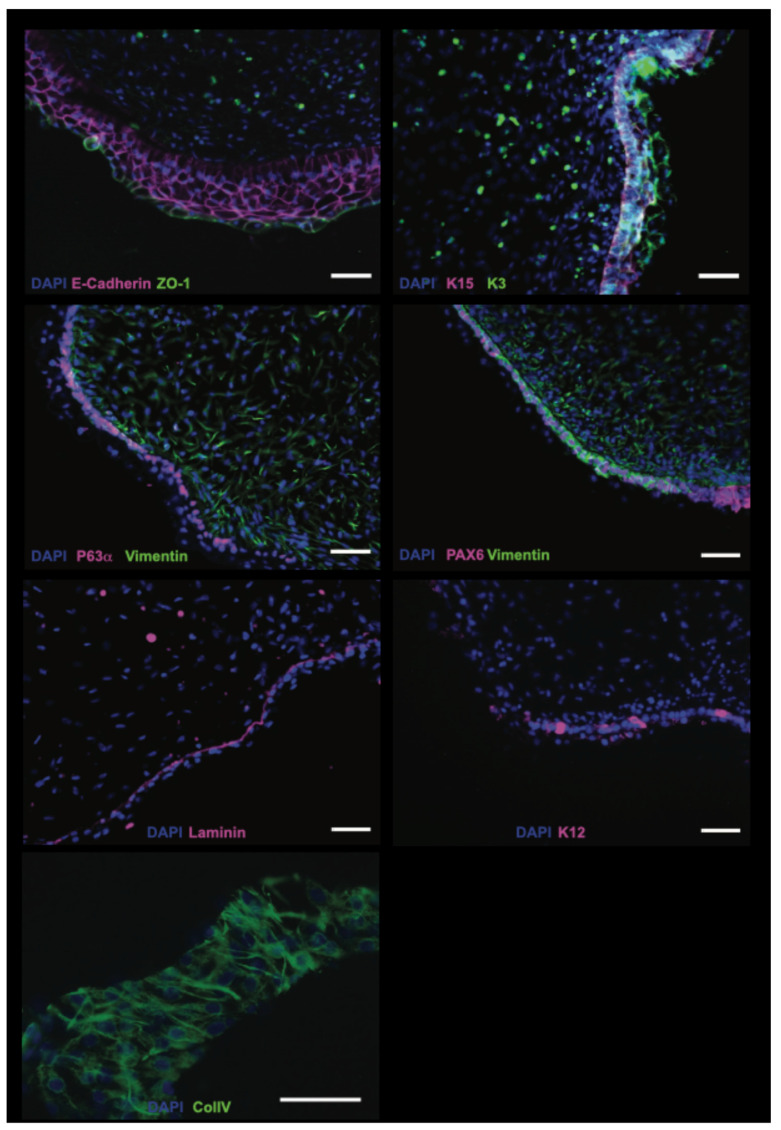
Immunostaining confirms corneal marker expression in 3D SEAM corneal organoids. After day 60, corneal organoids were fixed and stained for markers of cell types in native cornea. K = keratin; ColIV = Collagen IV. Scale bar = 50 μm.

The human corneal surface expresses the tight junction-associated protein ZO-1 as well as the epithelium marker E-Cadherin. We therefore examined whether the surface of the transparent cells in the putative corneal organoids also exhibited this expression pattern. Organoid slices 14 μm thick were cut and stained. The outer cellular layer of the transparent regions of the organoids showed clear expression of ZO-1 and E-Cadherin, suggesting these cells in fact were the outermost corneal epithelial cells (Figure 2). E-Cadherin was also expressed in 3–4 layers underneath the outermost ZO-1 expressing layer, resembling the outer epithelial layers of the cornea. No cells in the center of the organoid expressed E-Cadherin.

The limbus is a reservoir of stem cells for the cornea. We ascertained whether the putative corneal organoids express markers of the limbus. Using ΔNp63α as a marker of limbal stem cells, we stained the organoids with antibodies to ΔNp63α. We found that cells at the border of the interior organoid and the epithelial layer were positive for ΔNp63α, suggesting limbal cells may be specified in the organoids as early as day 60 (Figure 2). Vimentin is a useful marker for stromal cells of the cornea. We therefore co-stained the eye field transcription factor PAX6 with anti-vimentin. We found that an intermediate cell layer was positive for vimentin and PAX6, again exhibiting a polarized cell and specifying layers between distinct cellular markers of the cornea.

Considering the staining patterns of the previous cornea markers, we were curious whether a prototypical Bowman’s membrane may be forming. Using antibodies specific to laminin-1, a useful marker for the membrane, we observed lamina a couple of cellular layers away from the organoid surface, suggesting that the putative corneal organoids may be developing a Bowman’s membrane. Collagen is a foundational matrix in the eye, acting as a signaling hub and structural scaffold for both the epithelium and endothelium (Figure 2).

Keratins play an important role in corneal epithelial physiology, including mechanical strength, protective barrier, tissue integrity, signaling, growth and apoptosis. As such, the keratin moieties found in the cornea are distinct. We evaluated the expression of keratins known to be expressed in the cornea. We found positivity to antibodies to Keratins 3, 12, and 15 present in the outermost layers of the transparent region of the organoids (Figure 2).

PAX6 is a fundamental transcription factor for many cell types of the eye, including the cornea. We therefore expected the expression of PAX6 in the outermost layers of the transparent region of the organoids. The co-staining of PAX6 with Vimentin, as a marker of the stroma, exhibited segregated staining, illustrating both the putative stromal and epithelial layers in predicted cornea orientation (Figure 2). Based on immunostaining, the organoids exhibited features resembling the three outer layers of the cornea: epithelium, Bowman’s membrane, and stroma, and were positive for hallmark proteins of the cornea.

### 3.2. Single Cell RNA-Seq Characterization of 3D Corneal Organoids

**Figure 3:**
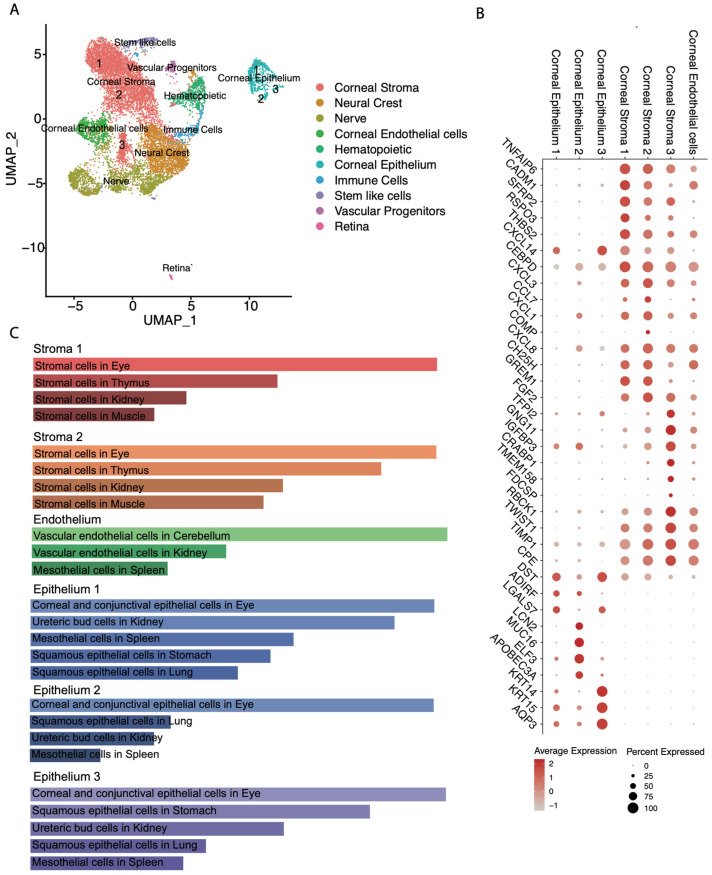
Single-cell RNA-seq identifies many cell types of the eye. (**A**) UMAP analysis of data identifies cellular clusters of the eye and relative expression proximity. (**B**) DotPlot of selected clusters composed of cornea cell types and the genes unique to each cluster. (**C**) Gene ontology of cornea cell types using Enrichr Descartes Cell Types and Tissue 2021.

To further characterize the 3D corneal organoids, we processed them for single-cell RNA-sequencing. We analyzed data by PCA and were able to detect 18 clusters. Positively expressed genes that were differentially expressed with a *p*-value < 0.05 were used in combination with Enricher GSEA and validated by Jensen Tissues, Mouse Gene Atlas and GO biological process terms to annotate the cluster identity. UMAP analysis was graphed and presented with the predicted cell annotation using Enricher GSEA (Figure 3A). Included in these organoids are putative retina, vascular progenitors, stem cell-like cells, immune cells, corneal stroma, corneal endothelium, and neural and neural crest progenitors.

Zooming in on cornea-specific populations, we generated a DotPlot using genes uniquely expressed in only one cluster (Figure 3B). We identified three corneal stroma clusters that expressed disparate genes involved in inflammation and immunity, and an endothelial cell population uniquely expressed and involved in cell adhesion, retinoic acid signaling and migration. Corneal epithelium clusters 1, 2, and 3 exhibit gene expression typical of corneal epithelial cells. There are genes shared among these clusters including PAX6, AQP3, CDH1, KRT5, KRT14, KRT15, MUC1, MUC4, SNCG, and TP63. KRT12 was unique in corneal epithelium 1. Corneal epithelium 2 was uniquely enriched in MUC16. MUC16 localizes to the tips of the microplicae or tiny folds on the corneal epithelium surface. Therefore, some corneal epithelium specialization seemed to occur in these organoids.

Using the top 250 genes for each population, we evaluated cell identity predictions using the Descartes cell types and tissue 2021 database, as it possesses a more comprehensive dataset for eye tissues than other datasets (Figure 3C). Each cornea cell subtype was predicted with the highest significance in all cases. For the stroma populations, stromal cells in the eye had a greater *p*-value > 1 × 10^−33^. Corneal endothelium was predicted to be vascular endothelial cells of the cerebellum, likely due to the absence of corneal endothelium in the dataset. All three corneal epithelium clusters were the top-predicted cell types, with *p*-value > 1 × 10^−15^.

**Figure 4:**
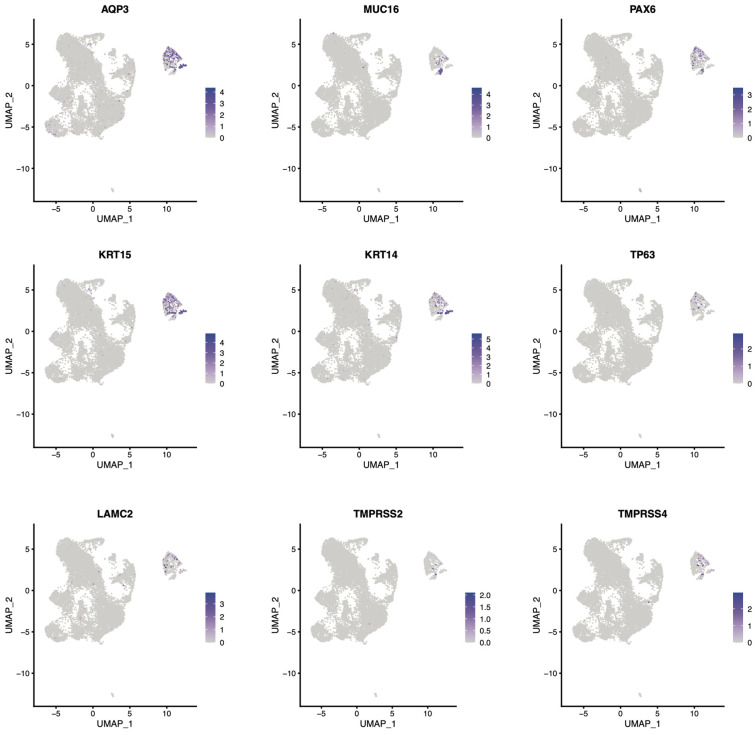
UMAP of corneal organoid genes representing essential cornea function. UMAP of genes related to cornea function demonstrates cell fate specification of the cornea and the utility of the organoids for studying cell fate specification, viral entry, cornea development, extracellular matrix formation, barrier function, fluid transport and more.

UMAP analyses of some of the identity genes for corneal epithelium were graphed to illustrate the divergent patterns of expression. Some genes were very tightly expressed solely in the corneal epithelium clusters, such as AQP3, PAX6, and Keratins (Figure 4). Others were found exclusively within individual epithelial clusters such as MUC16 or LAMC2. As noted in a previous publication using the SEAM whole-eye organoid culture protocol, TMPRSS4 is more heavily expressed in corneal epithelium than TMPRSS2 [16]. Observing this consistency between the two organoid protocols, including the generation of similar gene expression patterns within populations, is compelling and represents a meaningful validation. TP63 was found exclusively in corneal epithelium cluster 1, and KRT14 was absent in corneal epithelium cluster 2, but was present in corneal epithelium clusters 1 and 3 (Figure 4). How these patterns relate to adult corneal tissue subtypes will provide great insight into how these organoids diverge from native tissue and where they best resemble the native tissue. This information will be helpful to determine how these models are best utilized when exploring human physiology and disease.

**Figure 5:**
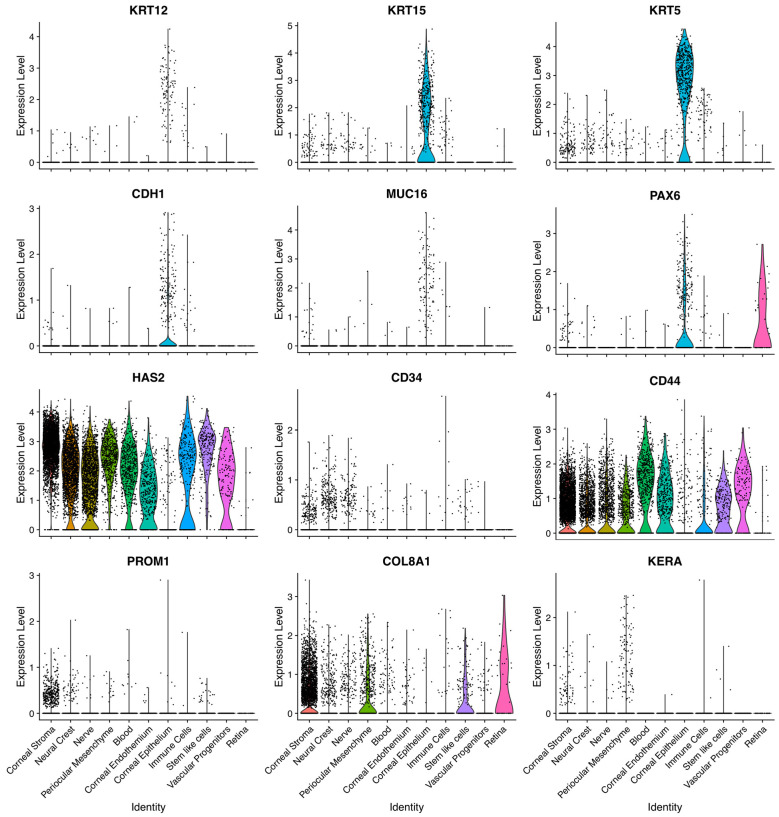
Violin plots illustrate the relative expression of selected genes involved in cornea cell function. Selected gene expression data graphed using violin plots exhibit the selectivity of some genes, such as those related to keratins, PAX6, CHD1 and MUC16, enriched in cornea epithelium cluster, while CD44, HAS2 and COL8A1 are more universal and make up the cornea extracellular matrix composition. KERA and PROM1 are expressed in corneal stroma.

Violin plots best illustrate the shape, density, and heterogeneity of gene expression distributions across cell populations—features that are biologically meaningful in single-cell data where cell-to-cell variability and subpopulation structure are central. Here, we graphed a selected set of genes expressed in this dataset to highlight some interesting details. Keratin 5 is much more highly expressed and more ubiquitously expressed among the whole corneal epithelium population than keratins 12 and 15, which may suggest a snapshot of the maturity stage of when these cells were taken (Figure 5). CDH1 expression is not homogenous, suggesting a still-developing epithelium. MUC16 is only expressed in a subset of epithelial cells. PAX6 expression is present in both corneal epithelium and retina specifying cells (Figure 5). Extracellular matrix-related proteins, including HAS2, CD34, CD44, and COL8A1, are found more homogenously across populations (Figure 5). PROM1, a gene involved in retinoic acid signaling and stem cell maintenance, is found in corneal stroma. KERA, a gene important for corneal clarity and collagen fibril organization, is found in corneal stroma (Figure 5).

## 4. Discussion

In this study, we developed and characterized a modified three-dimensional corneal organoid model derived from human embryonic stem cells using an adapted SEAM protocol. Our results demonstrate that these organoids successfully recapitulate key architectural and molecular features of the native human cornea, including stratified epithelial layers, putative limbal stem cell populations, stromal compartments, endothelium and a layer of laminin where a Bowman’s membrane would be. Through immunofluorescence staining and single-cell RNA sequencing analysis, we provide evidence that this 3D spheroid-based approach generates organoids with pertinent cellular diversity and spatial organization, signaling a valuable platform for studying corneal development, disease modeling, and therapeutic applications.

### 4.1. Development and Maturation Timeline of 3D Corneal Organoids

Our modified SEAM protocol successfully generated three-dimensional corneal organoids that undergo a predictable developmental trajectory over a 7-week culture period. The initial spheroid formation, followed by cyst development around day 13 and pigmentation around day 20, mirrors aspects of early ocular development, where neural crest and surface ectoderm interactions guide tissue specification [19]. The de-swelling observed between days 25 and 30 likely reflects maturation of the extracellular matrix and epithelial barrier function, consistent with the establishment of tight junctions and increased production of stromal components. Importantly, approximately 20% of organoids develop transparent regions—a critical indicator of corneal identity, as transparency is fundamental to corneal physiology and depends on precise collagen fibril organization, cellular arrangement, and absence of blood vessels.

The relatively modest success rate of ~20% for generating transparent organoids raises important considerations for protocol optimization. This variability likely reflects the stochastic nature of self-organization in organoid systems and the sensitivity of corneal fate specification to microenvironmental cues. The co-development of pigmented regions alongside transparent areas within individual organoids suggests that multiple ocular lineages can emerge simultaneously, consistent with the SEAM model’s capacity to generate both anterior (cornea, lens) and posterior (retina, RPE) ocular structures. Future iterations of this protocol may benefit from incorporating defined patterning factors or mechanical cues to enhance the efficiency and reproducibility of corneal organoid formation.

### 4.2. Cellular Architecture and Molecular Identity

Immunofluorescence analysis revealed that transparent regions of our organoids express key corneal markers in spatially appropriate patterns. The expression of ZO-1 and E-cadherin in the outermost cellular layer, with E-cadherin extending into 3–4 underlying layers, demonstrates proper epithelial stratification and the establishment of intercellular junctions essential for barrier function. This organization is critical for corneal physiology, as the multilayered epithelium provides the first line of defense against pathogens and maintains ocular surface homeostasis.

The detection of P63α-positive cells at the interface between the interior organoid and epithelial layer is particularly significant, as P63α is a well-established marker of limbal stem cells that maintain corneal epithelial renewal throughout life [20]. This spatial localization suggests that our organoids may be establishing a stem cell niche analogous to the limbal region in native corneas. The presence of vimentin-positive cells interior to the P63α layer, characteristic of corneal stromal keratocytes, further supports the notion that these organoids are developing distinct cellular compartments with appropriate anteroposterior polarity [21]. This polarized organization is essential for corneal function and represents a significant advance over two-dimensional culture systems that lack this spatial complexity.

Laminin-1 was detected several cell layers beneath the surface, located in orientation to the stroma and cornea epithelium in a manner resembling the location of a Bowman’s membrane. In native corneas, Bowman’s membrane is an acellular layer of collagen fibrils that provides structural support and is thought to play roles in wound healing and pathogen resistance [22]. While the presence of laminin-1 alone is not definitive proof of Bowman’s membrane formation, it indicates that the organoids are beginning to develop organized extracellular matrix structures characteristic of mature corneal tissue. Future studies employing transmission electron microscopy would be valuable to confirm the ultrastructural organization of this region.

The expression of cornea-specific keratins (K3, K12, and K15) in the outermost layers provides strong molecular evidence for corneal epithelial identity. Keratins 3 and 12 are particularly notable as they form the unique keratin pair expressed in differentiated corneal epithelial cells but not in other stratified epithelia [23]. The expression of K15, a marker associated with limbal stem cells, further supports the presence of a putative stem cell compartment [24]. The co-expression of PAX6, a master regulator of ocular development, with vimentin in segregated patterns demonstrates that the organoids are establishing distinct epithelial and stromal lineages, a fundamental feature of corneal tissue architecture.

### 4.3. Single-Cell Transcriptomic Insights into Cellular Diversity

Single-cell RNA sequencing provided a comprehensive molecular map of cellular diversity within our corneal organoids. The identification of 18 distinct clusters revealed that these organoids contain not only corneal cell types but also other ocular lineages, including putative retina, vascular progenitors and neural crest progenitors. This cellular heterogeneity reflects the pluripotent origins of the organoids and the self-organizing capacity of the SEAM protocol to generate multiple ocular fates. While this diversity presents challenges for applications requiring pure corneal tissue, it also offers opportunities to study cell–cell interactions and paracrine signaling events that occur during ocular development.

The identification of distinct corneal epithelium subclusters (1, 2, and 3) with shared and unique gene expression patterns provides insight into the differentiation states present in the organoids. All three clusters express canonical corneal markers, including PAX6, AQP3, CDH1, multiple keratins (K5, K14, and K15), mucins (MUC1 and MUC4), and TP63, thus confirming their corneal epithelial identity. However, the unique expression of KRT12 in epithelial cluster 1, MUC16 enrichment in epithelial cluster 2, and the presence of TP63 exclusively in epithelial cluster 1 suggest that these subclusters represent different stages of epithelial differentiation or regional specialization within the organoid epithelium.

The selective expression of MUC16 in epithelial cluster 2 is particularly noteworthy, as MUC16 localizes to the microplicae on the apical surface of corneal epithelial cells in vivo and plays critical roles in lubrication and barrier function [25]. This suggests that cluster 2 may represent the most superficial or functionally mature epithelial population. The restriction of TP63 expression to cluster 1, combined with the presence of KRT14 in clusters 1 and 3 but not 2, suggests that cluster 1 may contain progenitor or basal epithelial cells, while cluster 2 represents more differentiated superficial cells. This interpretation is consistent with the native corneal epithelium, where TP63-positive basal cells give rise to suprabasal and superficial cells that progressively differentiate [26]. Additionally, we observe that epithelial cluster 3 is high in basement membrane adhesion-related genes, LAMC2 (laminin production), ITGA6 (laminin-binding integrin) and DST, suggesting that this subcluster represents more basal corneal epithelium cells.

The identification of two distinct stromal clusters with differential expression of inflammatory and immunity-related genes indicates functional specialization within the stromal compartment. In native corneas, stromal keratocytes maintain quiescence but can become activated in response to injury or inflammation, transitioning to fibroblastic or myofibroblastic phenotypes [27]. The presence of stromal subpopulations with distinct inflammatory signatures may reflect this plasticity or suggest that the organoids contain stromal cells at different activation states. The detection of PROM1 expression in corneal stroma is intriguing, as PROM1 (CD133) is associated with stem cell maintenance and retinoic acid signaling, potentially indicating a progenitor population within the stromal compartment [28].

The corneal endothelium cluster, characterized by genes involved in cell adhesion, retinoic acid signaling, and migration, represents a crucial cell type that maintains corneal hydration and transparency in vivo through active fluid transport. While the Descartes database predicted this cluster as vascular endothelial cells of the cerebellum (likely due to the absence of corneal endothelium in the reference dataset), the expression profile and developmental context strongly support corneal endothelial identity. The presence of all three major corneal cell types—epithelium, stroma, and endothelium—demonstrates that these organoids resemble the full thickness of the cornea, with the layered organization of native tissue.

### 4.4. Gene Expression Patterns and Comparison to Native Tissue

The UMAP and violin plot analyses revealed important insights into the maturity and fidelity of gene expression in our organoids. The expression of KRT5 across the corneal epithelium population, with higher and more ubiquitous expression than KRT12 and KRT15, suggests that epithelial differentiation may not be fully complete by days 70–80. In mature human cornea, KRT12 is robustly expressed in suprabasal and superficial epithelial cells, while KRT5 is more restricted to basal layers. The relatively lower and more restricted expression of KRT12 in our organoids may indicate that additional maturation time or specific differentiation cues are needed to achieve the full differentiation program seen in adult corneal epithelium, as has been seen in older corneal organoids [7,8].

The expression of extracellular matrix-related genes, including HAS2, CD34, CD44, COL8A1, and KERA across multiple cell populations, demonstrates active-matrix production and remodeling. HAS2 (hyaluronan synthase 2) produces hyaluronic acid, a critical component of the corneal stroma that maintains hydration and tissue architecture [29]. The expression of KERA (keratocan), a cornea-specific keratan sulfate proteoglycan essential for maintaining collagen fibril organization and corneal transparency, in corneal stroma clusters is particularly encouraging [30]. The presence of COL8A1, a component of Descemet’s membrane produced by corneal endothelial cells, further supports the presence of functional endothelial populations [31].

The observation that TMPRSS4 is more heavily expressed than TMPRSS2 in the corneal epithelium, consistent with findings from other SEAM-based protocols, validates the reproducibility of gene expression patterns across two- and three-dimensional protocol variations [16]. These transmembrane serine proteases play roles in epithelial cell physiology and viral entry, and their expression patterns may have implications for modeling corneal viral infections and developing antiviral therapies.

### 4.5. Comparison to Existing Corneal Organoid Models

Our 3D spheroid-based approach represents an evolution of the original two-dimensional SEAM protocol, combining the advantages of three-dimensional culture with the established capacity of the SEAM method to generate multiple ocular cell types. Compared to the original Foster et al. [4] corneal organoids, which focused on generating multilayered structures mimicking epithelial, stromal, and endothelial layers through directed differentiation, our approach leverages self-organization principles to achieve similar outcomes with potentially improved cellular diversity.

Recent advances in corneal organoid technology have demonstrated applications in disease modeling, particularly in keratoconus, where single-cell RNA sequencing revealed dysregulated cell fates [8], and in regenerative medicine, where iPSC-derived corneal epithelial sheets have been used for clinical transplantation in limbal stem cell deficiency [12,13]. Our model contributes to this rapidly evolving field by providing a complementary approach that may be particularly well-suited for studying early developmental processes and cell fate specification events that occur during corneal organogenesis.

## 5. Limitations and Future Directions

Several limitations of our current model should be acknowledged and addressed in future studies. First, the relatively low efficiency of transparent organoid formation (~20%) limits throughput and increases variability between experiments. Optimization of culture conditions, including medium composition, growth factor concentrations, oxygen tension, and mechanical stimulation, may improve consistency and yield. The incorporation of additional patterning factors or temporal modulation of signaling pathways could potentially enhance the specification of corneal fate and reduce the formation of non-corneal cell types.

Second, functional characterization of the organoids is limited in the current study. While we have demonstrated expression of appropriate markers and established cellular diversity through transcriptomic analysis, we have not yet assessed whether these organoids possess functional properties of native cornea, such as barrier function, mechanosensation, innervation, or the ability to respond appropriately to injury. Future studies should incorporate functional assays, including barrier integrity measurements, evaluation of tight junction function, wound healing assays, and assessment of responses to inflammatory stimuli or pathogen challenge.

Third, the organoids analyzed in this study were harvested at days 70–80, but the full developmental trajectory and optimal harvest time for different applications remain to be determined, as done previously [7]. Longitudinal studies tracking organoid development over extended time periods, combined with temporal transcriptomic profiling, would provide valuable insights into maturation kinetics and identify the optimal time points for different experimental applications. Such studies could also reveal whether organoids eventually achieve more complete maturation with extended culture or whether they reach a developmental plateau that requires additional interventions to overcome.

## 6. Conclusions

We have successfully developed a three-dimensional corneal organoid model that recapitulates key features of human corneal tissue, including stratified epithelium, putative limbal stem cells, stromal cells, and endothelium, organized in a spatially appropriate manner. Through comprehensive characterization using immunofluorescence and single-cell RNA sequencing, we demonstrate that these organoids express appropriate corneal markers and exhibit cellular diversity comparable to developing corneal tissue. While further optimization is needed to improve efficiency, maturity, and functional properties, this model represents a valuable addition to the toolkit of in vitro systems for studying corneal biology and disease. As the field of organoid technology continues to advance, refinements to culture methods, integration with bioengineering approaches, and application to patient-specific iPSC lines will likely expand the utility of corneal organoids for both basic research and translational applications in corneal regenerative medicine.

## Data Availability

The original contributions presented in this study are included in the article/Supplementary Materials. Further inquiries can be directed to the corresponding author.

## Supplementary Materials

The following supporting information can be downloaded at: https://www.mdpi.com/article/10.3390/bioengineering13050518/s1, Table S1: SEAM Corneal Organoid Cluster Gene lists.
